# Supplementary material for: Re-Use of Caco-2 Monolayers in Permeability Assays—Validation Regarding Cell Monolayer Integrity
Source: Pharmaceutics. 2021 Sep 26;13(10):1563. doi: 10.3390/pharmaceutics13101563 (PMC8537988; doi:10.3390/pharmaceutics13101563)
Supplement: Supplementary file 1 [file pharmaceutics-13-01563-s001.zip › pharmaceutics-1360257-supplementary.pdf]

pharmaceutics

MDPI

Supplementary Materials: Re-Use of Caco-2 Monolayers in Permeability Assays—Validation regarding Cell Monolayer Integrity

Cristiana L. Pires, Catarina Praça, Patrícia A. T. Martins, Ana L. M. Batista de Carvalho, Lino Ferreira, Maria Paula M. Marques and Maria João Moreno

Section S1 Analytical procedure for LY quantification by HPLC

Lucifer Yellow was quantified by reversed-phase HPLC (Zorbax C18 column 250 × 4.6 mm, 5 µm particle size, 23 °C) with a fluorescence detector at λex/λem=430/530 nm. The mobile phase was ammonium formate 50 mM (pH 3.5 adjusted with formic acid) at a flow rate of 1 mL/ min. Typical chromatograms obtained for the injection of 100 µL (donor compartment) or 900 µL (acceptor compartment) of LY solutions in HBSS are shown in Figure S1 plot A. The calibrations curves obtained from the area between 5 and 10 min for 3 independent sets of solutions are shown in plots B1 and B2 for low and high LY concentrations respectively.

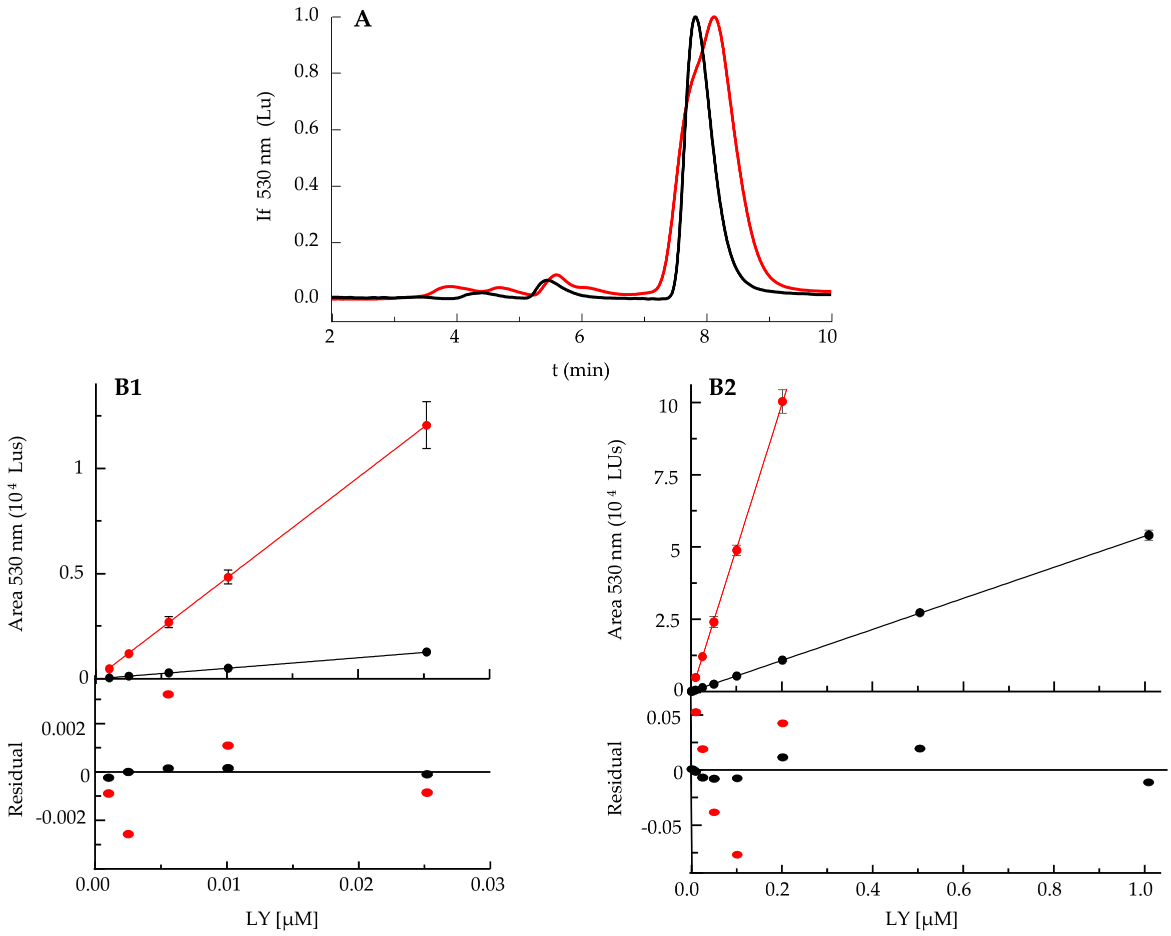

Figure S1. Typical chromatogram (A) of the fluorescence emission at 530 nm when excited at 430 nm obtained for LY, and calibration curves (B) for injections of 100 µL (black) or 900 µL (red). For concentrations lower than 0.03 µM (plot B1), the best linear fit has slope 5.05 (±0.01)×10^4, intercept 1.4 (±1.2) and r^2=0.9999 for 100 µL injections; and slope 4.79 (±0.01)×10^5, intercept 8 (±16) and r^2=0.9999 for 900uL injections. For concentrations up to 1 µM (plot B2), the best linear fit has slope 5.37 (±0.01)×10^4, intercept -16 (±37) and r^2=0.9999

for 100 μL injections; and 4.99 (±0.04)×10^5, intercept -715 (±429) and r^2=0.9998 for 900uL injections. The lower insets are the best fit residuals.

Section S2 - Statistical analysis of the effect of the different assay conditions

S2.1 - Effect of a single-time point permeability assay (60 min) on the cell monolayer TEER values

Characteristic values of the Transepithelial Electrical Resistance (TEER) before and after a LY permeability assay with a one-time sampling point at 60 min, for Caco-2 cell monolayers at different days after cell seeding. The very large number of assays performed allowed the characterization of the statistical distribution of TEER values, to obtain the characteristic average (μ) and standard deviation (σ) from which the confidence intervals at 95 % confidence (CI_{95}) were calculated. This was performed for μ and σ obtained directly from the TEER values, and from the parameters obtained for the best fit of a Normal distribution to the frequencies observed. Both statistical analysis lead to an underestimation of the lower limit of CI_{95} in some of the conditions analysed (lower than the lowest values obtained experimentally, values in grey) indicating that the distribution of TEER is skewed towards higher values. Given the expected LogNormal probability distribution of rate constants [1], and the inverse relation between TEER (a resistance) and the corresponding rate of ion permeation, the statistical analysis obtained from the best fit of a Normal distribution to Log(1/TEER) was also performed. This leads to an overestimation of the CI_{95} upper limits (higher than the highest values obtained experimentally, values in grey) indicating that the distribution of Log(1/TEER) is skewed towards lower values. This indicates that random variables other than the energy barrier for ion permeation are influencing the values measured for TEER, and that those are likely well described by a Normal distribution. Given the inadequacy of both statistical distributions to accurately describe the TEER variable, the simpler approach was selected, which is the calculation of μ and σ directly from the TEER values obtained experimentally (columns shaded in green).

Table S1. Characteristic TEER values and confidence intervals for the different cell monolayers before and after LY permeability assays with a 60 min sampling time point.

TEER values (kΩ cm²)		Directly from TEER			Best Fit Normal distribution to TEER values			Best Fit Normal distribution to Log(1/TEER)	
Day		μ	σ	CI_{95} [a]	μ	σ	CI_{95} [a]	μ	CI_{95} [a]
22	Before	1.0	0.5	0.1, 1.8	1.2	0.5	0.3, 2.0	0.9	0.3, 2.2
N=34	After	0.7	0.4	0.1, 1.3	0.7	0.2	0.4, 1.1	0.6	0.3, 1.4
25	Before	1.2	0.6	0.2, 2.1	1.0	0.4	0.3, 1.7	1.1	0.4, 2.6
N=29	After	0.9	0.5	0.1, 1.6	0.9	0.5	0.1, 1.7	0.8	0.3, 2.0
28	Before	1.1	0.4	0.5, 1.8	1.0	0.3	0.5, 1.5	1.1	0.6, 1.9
N=24	After	1.0	0.3	0.5, 1.6	1.1	0.2	0.7, 1.5	1.0	0.5, 2.0
All	Before	1.1	0.5	0.2, 1.9	1.0	0.5	0.2, 1.7	1.1	0.4, 2.3
N=87	After	0.8	0.4	0.2, 1.5	0.9	0.4	0.3, 1.6	0.8	0.3, 1.8

[a] lower and upper limits of the CI_{95} that are outside than the range of values obtained experimentally are shown in grey.

Some representative results obtained for individual cell monolayers are shown in Figure S2. The TEER values before the permeability assay are shown in grey, and those after are shown in white. For a small number of cell monolayers it is observed a small increase in TEER after the execution of the permeability experiment, but for most cell monolayers a decrease in TEER is observed. This decrease is usually small (less than 20 % variation), although a large decrease is observed in 1/3 of the cell monolayers. Incubation in culture media for two full days leads to the complete recovery of the TEER values pre-assay which usually become even larger than the value observed before the permeability assay. This over-recovery is frequently observed between day 22 to 25, while between day 25 and 28 the behaviour observed for the individual cell monolayers is very diverse (from small increases to a significant decrease). In all cell monolayers analysed, the TEER value observed before and after the permeability assay is always equal to or higher than 200 Ω cm^2, which is the value usually considered for tight Caco-2 cell monolayers [2,3].

The values obtained for the permeability coefficient of LY (P_{app}) are also shown in Figure S2. No systematic dependence is observed between P_{app} and TEER. This is the case for each day post-seeding when comparing distinct cell monolayers and for a given cell monolayer along the days post-seeding. This suggests that the high variability observed for the TEER values is not directly related with cell monolayer integrity. Among the factors that may influence the value of TEER, the electrode position relative to the cell monolayer may be of high relevance. It should be noted that while the TEER measurement is performed in a single location, LY permeability assay reflects the properties of the whole cell monolayer.

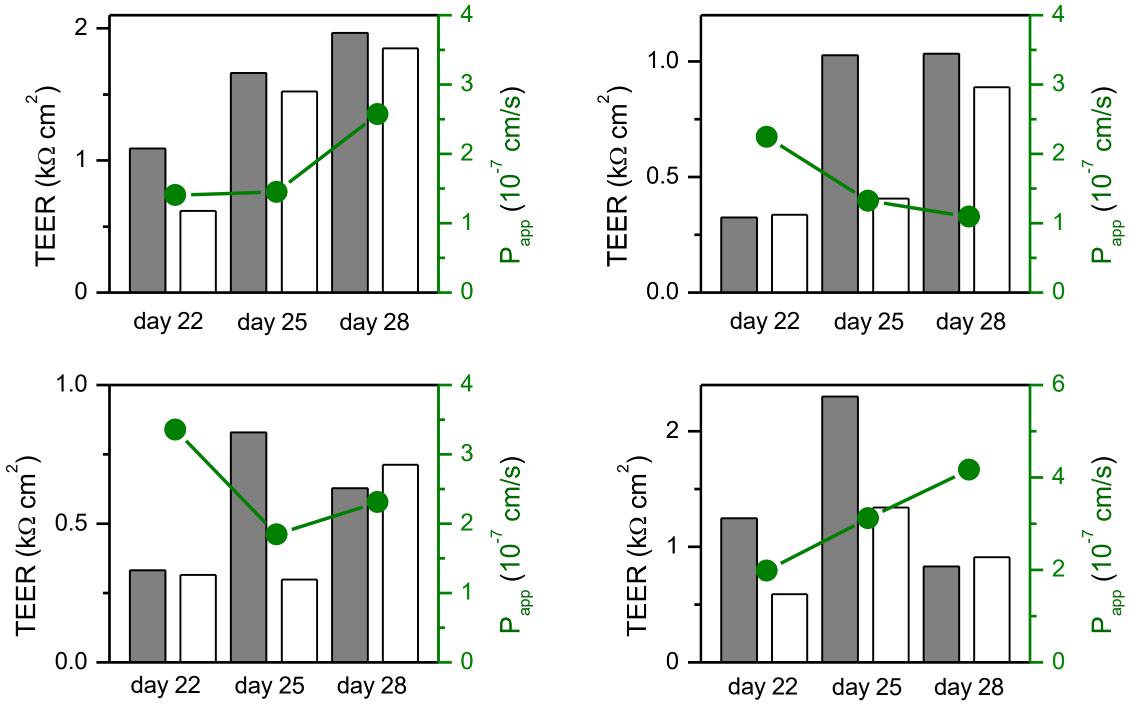

Figure S2. Representative results obtained for individual cell monolayers along days 22, 25 and 28 post-seeding, for the variation of their TEER values (before and after the LY permeability assays) and the permeability coefficient obtained for LY with a one-time sampling at 60 min.

S2.2 - Effect of cell monolayer re-use on LY Papp for a single-time point sampling (60 min)

Characteristic values of the apparent Permeability Coefficient of Lucifer Yellow (LY P_{app}) obtained for a one-time sampling point at 60 min, through Caco-2 monolayers at different days after cell seeding. The very large number of assays performed allowed the characterization of the statistical distribution of P_{app} values, to obtain the characteristic average (μ) and standard deviation (σ) from which the confidence intervals at 95 % confidence (CI_{95}) were calculated. This was performed for μ and σ obtained directly from the P_{app} values, and from the parameters obtained for the best fit of a Normal distribution to the frequencies observed. Both statistical analysis lead to an underestimation of the lower limit of CI_{95} in all conditions analysed (lower than the lowest values obtained experimentally, values in grey) indicating that the distribution of P_{app} is skewed towards higher values. Given the expected LogNormal probability distribution of rate constants [1], the statistical analysis obtained from Log(P_{app}) was also performed, leading to an adequate statistical description in all cases. For the experimental conditions were a large set of data was available (N ≥ 18) the statistical parameters obtained from the best fit of a Normal distribution to Log(P_{app}) are also shown. The results obtained where very similar to those obtained directly from Log(P_{app}). The simpler approach was therefore selected, with the calculation of μ and σ directly from the Log of the values of P_{app} obtained experimentally (columns shaded in green).

Table S2. Characteristic P_{app} values and confidence intervals at 95 % confidence (CI_{95}) obtained directly from the results and from the best fit of a Normal distribution to the frequency distribution of the Papp or Log(P_{app}) values. The numbers in grey correspond to limits of the confidence intervals that are outside the range of experimentally obtained values. Permeability coefficients where obtained for LY with a single sampling time point at 60 min.

Day	Conditions (number of experiments)	LY P_{app} (10^{-7} cm/s) Directly from P_{app}			Best Fit to P_{app}			Directly from Log(P_{app})		Best Fit to Log(P_{app})	
		μ	σ	CI_{95} [a]	μ	σ	CI_{95} [a]	μ	CI_{95} [a]	μ	CI_{95} [a]
22	all (35)	2.3	0.8	0.9, 3.6	2.0	0.7	0.9, 3.2	2.1	1.2, 3.9	2.1	1.1, 4.0
25	All (29)	1.9	0.7	0.8, 3.1	1.7	0.5	0.9, 2.5	1.8	1.0, 3.2	1.7	1.0, 2.9
	Single use (6)	2.1	1.1	-0.1, 4.2				1.8	0.6, 5.3		
	Re-use (23)	1.9	0.5	0.9, 2.8	1.7	0.4	1.1, 2.4	1.8	1.1, 2.6	1.7	1.1, 2.6
28	All (24)	2.2	1.2	0.1, 4.2	1.8	1.0	0.2, 3.4	1.9	0.8, 4.7	1.8	0.8, 4.2
	Single use (6)	3.3	1.2	1.0, 5.6				3.2	1.7, 6.1		
	Re-use (18)	1.8	0.9	0.2, 3.4	1.4	0.6	0.4, 2.4	1.6	0.7, 3.6	1.5	0.6, 3.5
All days	All (88)	2.1	0.9	0.6, 3.6	1.9	0.7	0.7, 3.0	2.0	1.0, 3.8	1.9	1.0, 3.9
	Single use (47)	2.4	0.9	0.8, 4.0	2.1	0.8	0.8, 3.5	2.1	1.2, 3.9	2.1	1.1, 4.0
	Re-use (41)	1.8	0.7	0.6, 3.1	1.6	0.5	0.8, 2.4	1.7	0.9, 3.2	1.7	0.9, 3.0

[a] lower and upper limits of the CI_{95} that are outside than the range of values obtained experimentally are shown in grey.

S2.3 - Effect of single and multi-time sampling on the cell monolayer TEER values

Table S3. Characteristic TEER values (in kΩ cm[2]) and confidence intervals for the different cell monolayers before and after LY permeability assays with a single (60 min) or multi (10, 20, 30, 60) sampling time points. The statistical analysis was performed directly from the TEER values obtained.

Day	Conditions (number of experiments)		TEER values (kΩ cm²) min	max	μ	σ	CI_{95} [a]
all	all (153)	Before	0.2	2.3	1.1	0.5	0.3, 1.9
		After	0.2	1.8	0.9	0.4	0.3, 1.5
22	all (62)	Before	0.3	2.0	1.0	0.4	0.3, 1.7
		After	0.3	1.6	0.8	0.4	0.2, 1.4
	one-time (34)	Before	0.3	2.0	1.0	0.5	0.1, 1.8
		After	0.3	1.6	0.7	0.4	0.1, 1.3
	multi-time (28)	Before	0.4	1.7	1.1	0.3	0.5, 1.7
		After	0.4	1.5	0.9	0.3	0.4, 1.5
25	all (51)	Before	0.2	2.3	1.0	0.5	0.2, 1.9
		After	0.2	1.8	0.9	0.4	0.2, 1.6
	single use all (12)	Before	0.2	1.8	1.0	0.5	0.1, 1.9
		After	0.3	1.7	1.0	0.4	0.2, 1.8
	single use, one-time (6)	Before	0.2	1.5	0.8	0.6	-0.2, 1.9
		After	0.3	1.3	0.8	0.4	0, 1.6
	single use, multi-time (6)	Before	0.4	1.8	1.1	0.4	0.2, 1.9
		After	0.8	1.7	1.2	0.4	0.5, 1.9
	re-use all (39)	Before	0.2	2.3	1.1	0.5	0.2, 2.0
		After	0.2	1.8	0.9	0.4	0.2, 1.6
	re-use, one-time (23)	Before	0.4	2.3	1.2	0.5	0.3, 2.2
		After	0.2	1.8	0.9	0.5	0, 1.7
	re-use, multi-time (16)	Before	0.2	1.6	0.8	0.4	0.1, 1.5
		After	0.5	1.5	0.9	0.3	0.4, 1.4
28	all (40)	Before	0.5	2.0	1.2	0.4	0.5, 1.9
		After	0.2	1.8	1.0	0.4	0.3, 1.6
	single use all (12)	Before	0.5	1.9	1.3	0.4	0.5, 2.0
		After	0.2	1.5	0.8	0.4	0.2, 1.5
	single use, one-time (6)	Before	0.5	1.5	1.2	0.4	0.5, 2.0
		After	0.2	1.3	0.8	0.4	0.1, 1.6
	single use, multi-time (6)	Before	0.9	1.9	1.3	0.4	0.4, 2.2
		After	0.5	1.5	0.8	0.4	0.1, 1.6
	re-use all (28)	Before	0.5	2.0	1.2	0.4	0.4, 2.0
		After	0.5	1.8	1.0	0.4	0.4, 1.6
	re-use, one-time (18)	Before	0.6	2.0	1.1	0.4	0.4, 1.7
		After	0.6	1.8	1.1	0.3	0.6, 1.6
	re-use, multi-time (10)	Before	0.5	1.9	1.4	0.5	0.6, 2.3
		After	0.5	1.7	0.9	0.5	0.1, 1.7

[a] lower and upper limits of the CI_{95} that are outside than the range of values obtained experimentally are shown in grey.

S2.4 - Effect of cell monolayer re-use and multi-time sampling on LY transport

Table S4. Statistical analysis of LY Permeability (P_{app}) and amount of LY transported into the acceptor compartment (Q_A (%)) for all the conditions tested. The average and standard deviation, and the corresponding confidence intervals at 95 % confidence (CI_{95}), were obtained directly from the analysis of Log(P_{app}) or Log(Q_A). The numbers in grey correspond to limits of the confidence intervals that are outside the range of experimentally obtained values.

Assay Conditions			LY P_{app} (10^{-7} cm/s)		LY Q_A (%)	
**Day**	**Sampling (N)**	**Monolayer use**	**μ**	**CI_{95}**	**μ**	**CI_{95}**
all	all (346)	Single use & re-use	7.0	1.4, 34.1	0.23	0.09, 0.61
	all 1st (153)		4.0	0.8, 19.8	0.19	0.08, 0.44
	1st 60 min (88)		2.0	1.0, 3.8	0.20	0.10, 0.39
	1st 10 min (65)		10.5	3.8, 29.1	0.18	0.06, 0.49
	2nd 10 min (66)		12.8	4.4, 36.7	0.21	0.07, 0.62
	3rd 10 min (62)		14.9	5.9, 38.1	0.25	0.10, 0.64
	4th 30 min (62)		7.0	2.7, 18.1	0.35	0.14, 0.91
22	all (144)	Single use	9.4	1.8, 47.7	0.30	0.13, 0.71
	all 1st (62)		5.0	0.9, 28.2	0.23	0.12, 0.43
	1st 60 min (35)		2.1	1.2, 3.9	0.21	0.12, 0.39
	1st 10 min (27)		14.9	7.6, 29.2	0.25	0.13, 0.49
	2nd 10 min (28)		17.6	8.0, 38.5	0.29	0.13, 0.65
	3rd 10 min (26)		22.0	11.1, 43.6	0.37	0.19, 0.72
	4th 30 min (28)		9.5	3.9, 23.0	0.48	0.20, 1.16
25	all (117)	Single use & re-use	7.3	1.6, 33.9	0.23	0.11, 0.50
	all 1st (51)		4.0	0.8, 20.7	0.18	0.10, 0.34
	1st 60 min (29)		1.8	1.0, 3.2	0.18	0.10, 0.32
	1st 10 min (22)		11.2	5.5, 22.9	0.19	0.09, 0.38
	2nd 10 min (22)		14.9	7.0, 32.0	0.25	0.12, 0.54
	3rd 10 min (22)		14.8	7.3, 30.0	0.25	0.12, 0.50
	4th 30 min (22)		7.1	3.9, 13.0	0.36	0.20, 0.66
	all 1st (12)	Single use	3.6	0.8, 16.3	0.15	0.06, 0.35
	1st 60 min (6)		1.8	0.6, 5.4	0.18	0.06, 0.54
	1st 10 min (6)		7.3	4.3, 12.4	0.12	0.07, 0.21
	2nd 10 min (6)		11.3	5.9, 21.8	0.19	0.10, 0.37
	3rd 10 min (6)		11.7	5.0, 27.6	0.20	0.08, 0.46
	4th 30 min (6)		6.1	3.6, 10.3	0.31	0.18, 0.52
	all 1st (39)	re-use	4.1	0.7, 23.3	0.20	0.12, 0.33

	1st 60 min (23)		1.8	1.1, 2.9	0.18	0.11, 0.29
	1st 10 min (16)		13.2	7.6, 35.8	0.22	0.13, 0.39
	2nd 10 min (16)		16.5	7.6, 35.8	0.28	0.13, 0.60
	3rd 10 min (16)		16.2	8.5, 31.0	0.27	0.14, 0.52
	4th 30 min (16)		7.5	3.9, 14.4	0.38	0.20, 0.73
	all (86)		4.1	1.3, 13.5	0.14	0.05, 0.37
	all 1st (40)		2.9	0.8, 10.4	0.14	0.05, 0.44
	1st 60 min (24)		1.9	0.8, 4.7	0.19	0.08, 0.47
	1st 10 min (16)	Single use & re-use	5.4	1.9, 15.7	0.09	0.03, 0.26
	2nd 10 min (16)		5.9	2.6, 13.6	0.10	0.04, 0.23
	3rd 10 min (14)		7.6	4.4, 13.1	0.13	0.07, 0.22
	4th 30 min (16)		4.1	1.7, 9.6	0.20	0.09, 0.48
	all 1st (12)		5.2	1.5, 18.8	0.22	0.07, 0.68
	1st 60 min (6)		3.2	1.7, 6.1	0.32	0.17, 0.62
28	1st 10 min (6)	Single use	8.7	2.5, 30.2	0.15	0.04, 0.51
	2nd 10 min (6)		8.2	3.0, 22.2	0.14	0.05, 0.37
	3rd 10 min (6)		8.7	4.8, 15.6	0.15	0.08, 0.26
	4th 30 min (6)		4.7	2.4, 9.3	0.24	0.12, 0.47
	all 1st (28)		2.2	0.8, 6.6	0.12	0.04, 0.33
	1st 60 min (18)		1.6	0.7, 3.6	0.16	0.07, 0.36
	1st 10 min (10)		4.1	2.0, 8.4	0.07	0.03, 0.14
	2nd 10 min (10)	re-use	4.9	2.6, 9.1	0.08	0.04, 0.15
	3rd 10 min (8)		6.8	4.0, 11.7	0.11	0.07, 0.20
	4th 30 min (10)		3.7	1.3, 10.2	0.19	0.07, 0.51

The average and standard deviation of the cumulative amount of LY that reaches the acceptor compartment is represented in Figure S3, for cell monolayers used on day 22, 25 and 28.

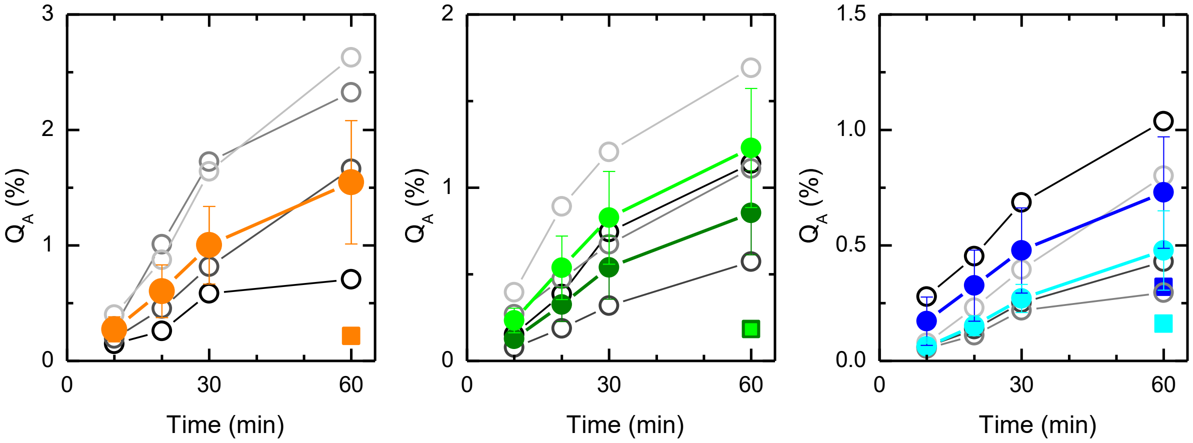

Figure S3. Cumulative amount of LY that reaches the acceptor compartment for permeability experiments performed with multi-time sampling (circles) or with a one-time sampling (squares) on cell monolayers at day 22 (left), 25 (middle) and 28 (right) post-seeding. On days 25 and 28, the results obtained for cell monolayers used in a single permeability experiment are shown in dark colours, and those obtained for re-used cell monolayers are in light colours. The average and standard deviation obtained for all assays in a given condition are shown as filled symbols and thick lines, and some representative results obtained for individual cell monolayers are shown by open symbols and thin lines.

S2.5 - Effect of cell batch, passage number and day post-seeding on TEER and LY transport

Table S5. Inter-variability between cell batches and passages regarding TEER values before the permeability assay and amount of LY transported during the first sampling interval (60 or 10 min for single or multi-time sampling, respectively).

Day	Cell conditions		Monolayer use	Sampling	Q_A (%)[a]		TEER (kΩ cm²)	
	Batch	Passage Number			μ	CI_{95}	μ	CI_{95}[b]
22	1	95	Single	One-time	0.18	0.12, 0.28	1.35	0.59, 2.11
	1	98		Multi-time	0.19	0.11, 0.31	1.10	-0.08, 2.29
	1	100			0.36	0.23, 0.56	1.06	0.63, 1.49
	1	104			0.16	0.07, 0.34	0.82	0.22, 1.42
	1	105			0.22	0.13, 0.36	1.27	0.80, 1.74
	2	95		One-time	0.27	0.19, 0.38	0.53	0.15, 0.90
	2	98			0.30	0.13, 0.72	0.67	-0.69, 2.02
	3	95			0.19	0.08, 0.43	0.87	0.18, 1.56
25	1	95	Single	One-time	0.13	0.04, 0.42	1.19	-0.15, 2.52
	1	95	Re-use		0.21	0.10, 0.44	2.04	1.55, 2.53
	1	98	Single	Multi-time	0.10	0.05, 0.21	1.31	0.33, 2.30
	1	100	Re-use		0.25	0.15, 0.43	0.80	-0.18, 1.77
	1	104	Single		0.14	0.08, 0.25	0.87	-0.05, 1.79
	1	105	Re-use		0.20	0.11, 0.38	0.84	0.30, 1.38
	2	95	Re-use	One-time	0.16	0.11, 0.22	0.96	0.67, 1.25
	2	98	Single		0.25	0.07, 0.87	0.51	-0.27, 1.28
	3	95	Re-use		0.19	0.14, 0.26	0.95	0.34, 1.57
28	1	95	Single	One-time	0.39	0.17, 0.90	1.35	1.03, 1.67
	1	95	Re-use		0.33	0.19, 0.59	1.59	0.04, 3.15
	1	98	Single	Multi-time	0.09	0.03, 0.26	1.27	0.19, 2.36
	1	100	Re-use		0.09	0.02, 0.38	1.42	0.44, 2.39
	1	104	Single		0.23	0.08, 0.69	1.29	0.02, 2.56
	1	105	Re-use		0.06	0.04, 0.09	1.44	0.43, 2.45
	2	95	Re-use	One-time	0.11	0.06, 0.22	0.98	0.62, 1.35
	2	98	Single		0.26	0.18, 0.37	1.11	-0.23, 2.45
	3	95	Re-use		0.17	0.10, 0.27	0.96	0.52, 1.41

[a] μ and CI_{95} calculated from the statistical parameters obtained for LogQ_A. [b] lower and upper limits of the CI_{95} that are outside than the range of values obtained experimentally are shown in grey.

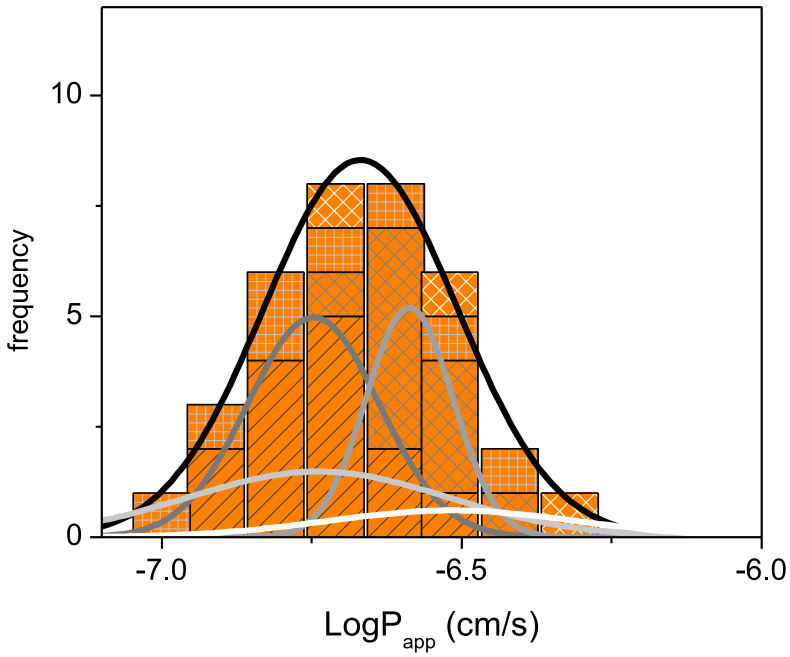

Figure S4. Distribution of LogP_{app} obtained on permeability assays with a single-time sampling (60 min) for cell monolayers from different batches and distinct passage numbers, at day 22 post-seeding: passage 95 with cell batch 1, ; passage 95 with cell batch 2, ; passage 95 with cell 3, ; and passage 98 with cell batch 2, . The lines are the best fit of a Normal distribution with the colours dark grey, grey, light grey and white, respectively. The black line is the best fit to the cumulative results at all cell passage numbers and batches.

S2.6 – Multivariate analysis

Table S6. Multivariate analysis (MVA) to evaluate the correlation between the variables TEER (before and after the permeability assay) or the amount of LY transported into the acceptor compartment (Q_A) with the distinct experimental variables. The results obtained when this statistical analysis was performed for P_{app} where equivalent to those obtained for Q_A.

Independent Variables considered	**TEER (Ω cm²)** before assay			**TEER (Ω cm²)** after assay			**Q_A (%)**			P_{app} (cm/s)		
	**Coefficient**	**CI95**	***P*-value**	**Coefficient**	**CI95**	***P*-value**	**Coefficient**	**CI95**	***P*-value**	**Coefficient**	**CI95**	***P*-value**
Cell batch	-317	-437, -196	**<0.0001**	-101	-205, 3	0.06	-2×10^{-2}	$(-5, 1)\times10^{-2}$	0.29	-4×10^{-9}	$(-1, 1)\times10^{-7}$	0.95
Cell passage	-12	-48, 23	0.49	-21	-49, 7	0.14	-3×10^{-3}	$(-1, 0.5)\times10^{-2}$	0.49	-3×10^{-8}	$(-6, 0.4)\times10^{-8}$	0.08
Day post-seeding	29	-6, 65	0.11	12	-17, 41	0.40	-7×10^{-3}	$(-2, 0.06)\times10^{-2}$	0.07	-7×10^{-8}	$(-1, -0.3)\times10^{-7}$	**0.0002**
Re-use	67	-110, 245	0.46	83	-59, 226	0.25	-3×10^{-2}	$(-7, 1)\times10^{-2}$	0.16	2×10^{-8}	$(-1, 2)\times10^{-7}$	0.78
Sampling time points	-162	-468, 144	0.29	141	-103, 386	0.26	-5×10^{-3}	$(-7,6)\times10^{-2}$	0.89	1×10^{-6}	$(0.9, 1)\times10^{-6}$	**<0.0001**
TEER before assay				0.28	0.15, 0.42	**<0.0001**	-2×10^{-5}	$(-6, 2)\times10^{-5}$	0.28	-8×10^{-11}	$(-2, 0.9)\times10^{-10}$	0.36
TEER after assay							-7×10^{-5}	$(-5, 4)\times10^{-5}$	0.77	5×10^{-11}	$(-2, 2)\times10^{-10}$	0.65

Data analysed corresponds to 152 permeability assays. CI_{95} is the confidence interval at 95 % confidence, *P*-values <0.05 are in bold.

Section S3 – Quantitative analysis of the monolayer images obtained by confocal microscopy

A very large number of cell monolayers (N=52) have been characterized by confocal fluorescence microscopy to evaluate the effect of the distinct conditions on the morphology of Caco-2 monolayers. The nuclei were stained with Hoechst 33342, and the tight junction protein ZO-1 was stained with rabbit polyclonal primary antibody and Cy3 goat anti-rabbit IgG. A quantitative analysis of the confocal images has been performed using the Image J software, and the corresponding average and standard deviation for the cell density and area occupied by nuclei and ZO-1 is shown in Table S7. A multivariate analysis was also performed for this set of data (including the cell batch and passage number, day post-seeding, cell monolayer use and sampling conditions, as well as the corresponding TEER values and amount of LY transported) and is shown in Table S8.

Effect of the number of days post-seeding. At day 22 post-seeding, a total of 7 independent cell monolayers were characterized by confocal microscopy before the execution of any permeability assay. It is observed that the number of nuclei in the area analysed (1.8×10^5 μm^2, typically in the central area of the filter, selected to be representative of the cell monolayer after visualization of the total area of the filter) varies significantly, being 570 ± 136, leading to a cell density of $3.1(\pm0.7)\times10^5$ cm^{-2}. The area occupied by the nuclei increases with the number of nuclei but not proportionally (the same behaviour is observed for the dependence of the fluorescence intensity from the stained nuclei due to the application of a threshold in the quantitative analysis of the image, to overcome differences in the staining efficiency). The non-proportionality between the area occupied and the number of nuclei indicates that the shape of the cell adjusts to the cell density in the monolayer, becoming wider or thicker in cell monolayers with lower or higher cell density respectively. A positive correlation is observed between the TEER value and the cell density ($R^2 = 0.27$, Figure S5), most likely due to an increase in the cell monolayer thickness.

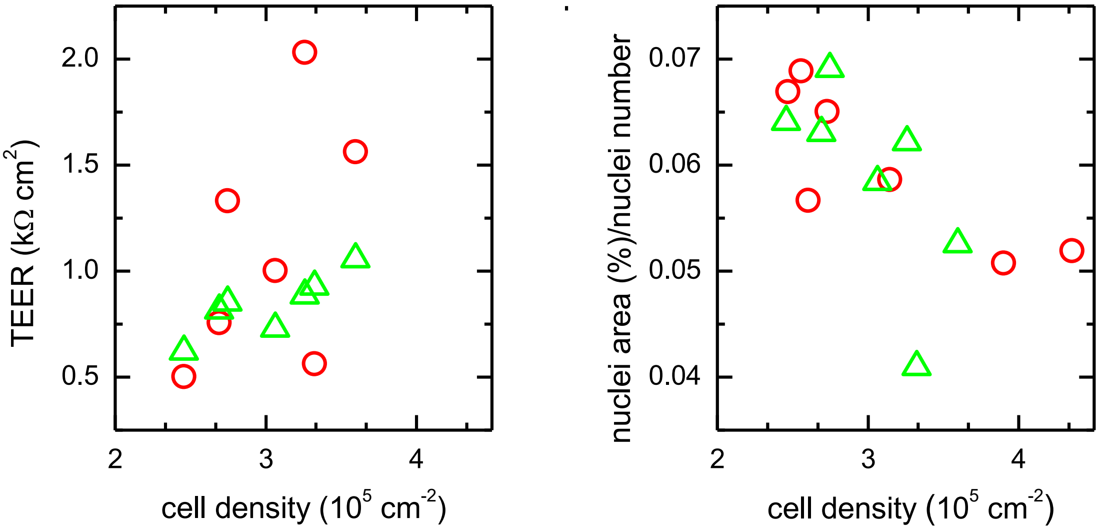

Figure S5. Correlation between the TEER value (left plot) and normalized nuclei area (right plot), with the cell density for monolayers on day 22 post-seeding, before (○) and after (△) the execution of a LY permeability assay.

The 3 cell monolayers characterized at day 25 post-seeding (that were not previously used in permeability assays, row single use/before) show a very small increase in the cell density (Δμ=4 %), essentially no variation in the area occupied by the nuclei (Δμ=-1 %), but a very significant increase in the area occupied by tight junctions (from stained ZO-1, (Δμ=80 %). At day 28, the cell density and the area occupied by the

nuclei in the 4 cell monolayers characterized is essentially the same observed on day22 and 25. The area occupied by tight junctions is higher than that observed at day 22, but smaller than at day 25.

The variations observed along the days post-seeding suggest that the expression of ZO-1 increases over time, which is consistent with the small decrease observed in LY permeation and the increase in TEER. However, the variations observed are relatively small when compared to the variability at a given condition, leading to differences that are not statistically significant (see Table S8).

Effect of the execution of a single permeability assay. The cell monolayers analysed after the execution of a LY permeability assay on day 22 post-seeding (3 for one-time and 4 for multi-time sampling) show morphological characteristics very similar to those observed for unused cell monolayers. For one-time sampling the cell density shows a small decrease which is not statistically significant and essentially no variation is observed in the cell monolayers subject to multi-time sampling. This indicates that the execution of the permeability assay does not lead to significant detachment of cells from the filter. As observed for unused cell monolayers, there is a strong correlation between the value of TEER measured after the permeability assay and the cell density ($R^2 = 0.7$). The area occupied by tight junctions is 9 % smaller in the cell monolayers used on one-time permeability assays, in agreement with the lower cell density. This variation is however smaller than the standard deviation observed in each condition and is therefore not statistically significant. When the cell monolayers from the same batch and passage number are compared, it is usually observed a small decrease in the cell density after the execution of the permeability assay (5 out of 7 independent sets), although a small increase is observed in two sets. A very high variability is observed in the area occupied by the tight junctions when pairs of monolayers are compared (increase or decrease) with the average values showing a small effect of the execution of the permeability assay. This highlights the difficulty in the quantification of the stained ZO-1, where a continuous network may be visible in the confocal images but which displays very large variations in the fluorescence intensity. This difficulty may be related with inhomogeneities in the cell monolayer staining, due to the slow dynamics of the strong interactions established (between the antibody and ZO-1, and between these and the fluorescent antibody).

When compared with cell monolayers on day 25 before the permeability assay, the confocal images of cell monolayers after execution of a single permeability assay with one-time sampling show a decrease in the number of nuclei ($\Delta\Delta\mu$=-33 %), in the area occupied by the nuclei ($\Delta\Delta\mu$=-15 %) and by tight junctions ($\Delta\Delta\mu$=-55 %). It should however be noted that although the execution of the permeability assay leads to a decrease in the area occupied by tight junctions, this parameter is still higher than that observed on day 22 post-seeding ($\Delta\mu$=5 %). Equivalent results are observed for multi-time sampling, and a very similar behaviour is observed for cell monolayers on day 28 post-seeding.

Globally, the results show that the execution of a permeability assay tends to decrease the number of cells in the monolayer and the area occupied by tight junctions, but that those effects are smaller than the variability within each condition, and thus not statistically significant.

Effect of cell monolayer re-use. The very high variability observed in the cell density and the relatively small number of cell monolayers characterized, does not allow the establishment of correlations between this morphological property and the re-use of the cell monolayer. Some tendencies are however observed which are discussed below. The cell density of the cell monolayers after two full days in culture media is usually similar to (or higher than) the density observed for cell monolayers at the same day post-seeding that were not previously used in permeability assays. This shows that the cell monolayer recovers from the small decrease observed after the execution of the permeability assay. The variations observed in the area occupied by

tight junctions are also very small and always within the variability observed within each set of conditions. An interesting observation is that the variations induced by the execution of the permeability assay seems to be smaller for re-used cell monolayers than for cells used on a single permeability experiment.

Effect of the sampling time points. Given the small decrease (although not statistically significant) observed in the cell density and area occupied by nuclei and tight junctions after the execution of the permeability assay, one could expect a higher effect for a larger number of sampling time points. This is not however observed in the cell monolayers characterized in this work. In fact, the effects observed when using multi-time sampling are actually smaller than when using only one-time sampling. The differences are however within the uncertainty of the variables and are not statistically significant.

Multivariate Analysis. The multivariate analysis performed on the set of cell monolayers characterized by confocal microscopy is shown in Table S8. All conditions have been included in the statistical analysis. No strong correlations were observed between the different parameters, with p-values being always larger than 0.05. Correlations with p-value < 0.2 are nevertheless identified and discussed below.

The number of nuclei in the area analyzed shows a positive correlation with the total area occupied by nuclei, and for cell monolayers used in permeability assays it also shows a positive correlation with the TEER value of the monolayer. Those correlations were already discussed above, when analyzing the results obtained for day 22. The MVA shows that a weak correlation is also observed for cell monolayers at all days post-seeding and all conditions of the permeability assay. No correlation was observed between the cell density and the TEER value before the permeability assay (including the cell monolayers characterized before the permeability assay). In fact, no correlation was also observed between TEER before and after the permeability assay, although a strong correlation is observed when the complete set of permeability experiments is considered (Table S6, $p<0.001$). This reflects the extremely large variability in the TEER value measured for the cell monolayers, that is statistically not well described by the relatively small set of cell monolayers characterized by confocal microscopy.

The area occupied by the nuclei shows the expected positive correlation with the cell density and a weak negative correlation with the cell passage number,

The area occupied by tight junctions (as seen by ZO-1 staining) shows a positive correlation with the sampling used in the permeability assay, being higher for cell monolayers used in multi-time sampling.

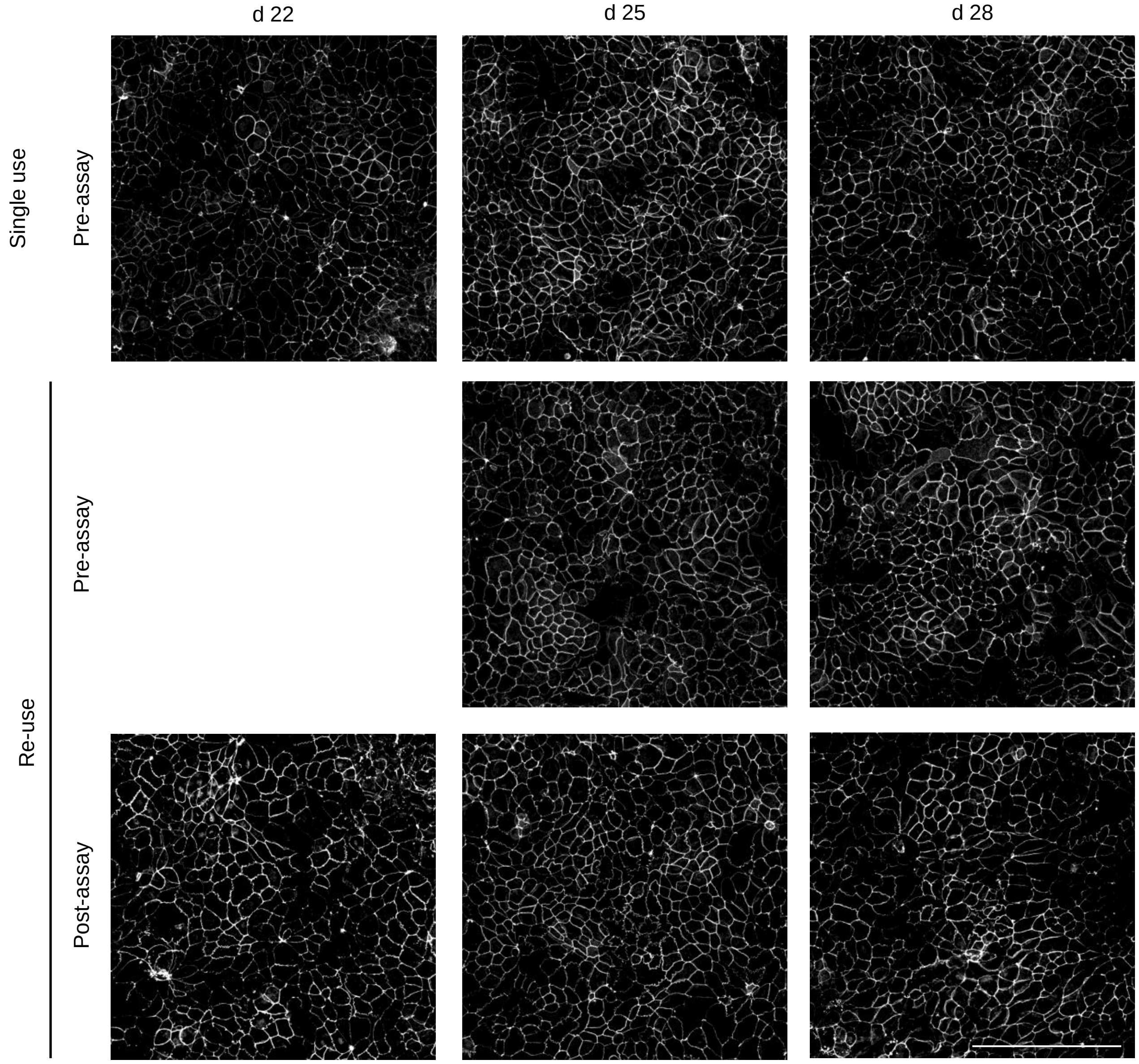
d 22
d 25
d 28
Single use
Pre-assay
Re-use
Pre-assay
Post-assay

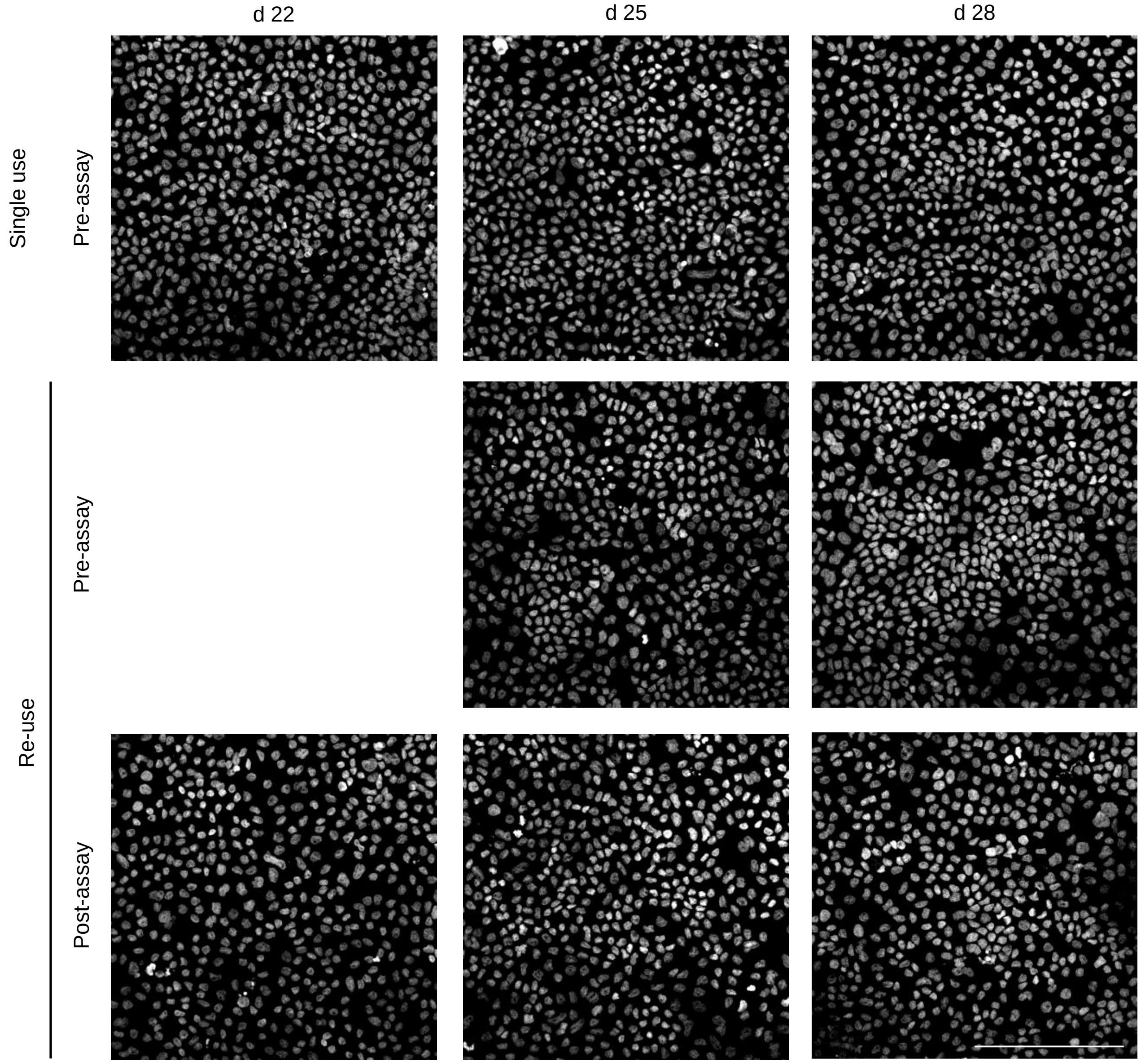

Figure S6. Confocal microscopy images of Caco-2 monolayers stained for ZO-1 (top picture) and nuclei (bottom picture). Representative images for cell monolayers at day 22, 25 and 28 are shown in the left, middle and right panels, respectively. In the upper plots the monolayer was not used in permeability assays, while in the additional plots the monolayers were previously used. The lower plots correspond to monolayers immediately after the LY permeability assay, and in the middle plots the images correspond to cell monolayers that were maintained in culture media for two days after the permeability assay. Images are a maximum intensity projection of 8-9 z-stacks, scale bar 200 μm.

Table S7. Cell density and fractional area occupied by nuclei (stained with Hoechst 33342), and by tight junctions (from ZO-1 staining with a rabbit polyclonal antibody and Cy3 goat anti-rabbit IgG), for images obtained by confocal microscopy (428×428 μm²) of cell monolayers in different conditions: not used on permeability assays, before re-use in permeability assays after incubation in culture media for two full days, and immediately after a permeability assay with one-time sampling (60 min) or multi-time sampling (10, 20, 30, and 60 min). The images where analysed using the software Image J. Each image was manually threshold to select nuclei or ZO-1 only fluorescence.

Day	Monolayer use	Permeability assay conditions (N)[a]	Nuclei						ZO-1		
			Cell density (x 10^5 cm^{-2})			Area (%)			Area (%)		
			μ ± σ	Δμ (%)[b]	ΔΔμ (%)[c]	μ ± σ	Δμ (%)[b]	ΔΔμ (%)[c]	μ ± σ	Δμ (%)[b]	ΔΔμ (%)[c]
22	Single	Before (7)	3.1 ± 0.7			33 ± 5			6 ± 2		
		One-time (3)	2.8 ± 0.4	-9	-9	34 ± 4	1	1	6 ± 2	-9	-9
		Multi-time (4)	3.2 ± 0.4	2	2	31 ± 4	-8	-8	7 ± 3	4	4
25	Single	Before (3)	3.2 ± 0.8	4	4	33 ± 7	-1	-1	12 ± 5	80	80
		One-time (2)	2.2 ± 0.1	-30	-33	28 ± 2	-15	-15	5 ± 2	-20	-55
		Multi-time (2)	2.7 ± 0.3	-13	-16	30 ± 5	-9	-9	7 ± 1	5	-42
	Re-use	Before One-time (3)	4.6 ± 2	48	63	38 ± 5	15	14	8 ± 2	31	44
		Before Multi-time (3)	3.0 ± 0.4	-4	-6	33 ± 3	-1	7	8 ± 1	28	23
		One-time (3)	4.5 ± 1	44	-3	39 ± 6	18	3	9 ± 3	43	9
		Multi-time (3)	3.0 ± 0.1	-4	1	32 ± 5	-3	-2	9 ± 2	33	4
28	Single	Before (4)	3.0 ± 0.1	-3	-7	32 ± 7	-4	-4	8 ± 2	25	-31
		One-time (2)	2.8 ± 0.1	-11	-8	34 ± 3	1	5	6 ± 2	1	-19
		Multi-time (2)	2.7 ± 0.3	-12	-10	29 ± 4	-14	-10	7 ± 1	7	-14
	Re-use	Before One-time (3)	3.3 ± 0.7	7	-26	39 ± 4	16	-2	6 ± 1	-11	-37
		Before Multi-time (3)	3.4 ± 0.6	8	12	35 ± 9	6	9	8 ± 2	30	-2
		One-time (2)	3.1 ± 0.6	1	-5	39 ± 10	18	2	5 ± 2	-28	-19
		Multi-time (3)	3.0 ± 0.1	-2	-9	33 ± 2	0	-6	8 ± 1	20	-8

[a] number of cell monolayers characterized. [b] Percent variation relative to the first condition analysed (day 22 post-seeding, before any permeability assay). [c] Percent variation relative to the condition immediately before (e.g. single use before day 25 *vs* single use before 22, one-time/multi-time *vs* before in the same condition, re-use one-time/multi-time at day 25 before *vs* after one-time/multi-time at day 22.

Table S8. Multivariate analysis (MVA) of the cell monolayers whose morphological characteristics have been characterized by confocal microscopy. The total number of cell monolayers in this analysis is 52, including cells from batches 1 and 2, in passage numbers 95 to 105, days post-seeding 22, 25 and 28 (single use and re-use), and one-time or multiple sampling (see Table S7 for the number of cell monolayers in each condition).

Independent Variables considered	TEER (Ω cm^2)						Q_A (%)			Cell density (cm^{-2})			Nuclei Area (%)			ZO-1 Area (%)		
	Before assay			After assay														
	**Coefficient**	**CI_{95}**	***P*-value**	**Coefficient**	**CI_{95}**	***P*-value**	**Coefficient**	**CI_{95}**	***P*-value**	**Coefficient**	**CI_{95}**	***P*-value**	**Coefficient**	**CI_{95}**	***P*-value**	**Coefficient**	**CI_{95}**	***P*-value**
Cell batch	-3×10^{2}	$(-9, 1)\times10^{3}$	0.19	-1×10^{2}	$(-6, 4)\times10^{2}$	0.64	-6×10^{-2}	$(-2, 1)\times10^{-1}$	0.50	-5×10^{4}	$(-1, 0.3)\times10^{5}$	0.23	-3	-9, 4	0.39	2	-2, 6	0.24
Cell passage	3×10^{1}	$(-3, 8)\times10^{2}$	0.37	-1×10^{1}	$(-8, 5)\times10^{1}$	0.75	1×10^{-2}	$(-1, 4)\times10^{-2}$	0.31	7×10^{3}	$(-0.4, 2)\times10^{3}$	0.20	-0.6	-1, 0.2	0.12	-0.2	-0.8, 0.3	0.45
Day post-seeding	5×10^{1}	$(-0, 1)\times10^{2}$	0.07	-1×10^{1}	$(-7, 5)\times10^{1}$	0.69	-1×10^{-3}	$(-2, 2)\times10^{-2}$	0.93	-6×10^{3}	$(-2, 0.4)\times10^{4}$	0.21	-1×10^{-2}	-0.8, 0.7	0.97	-0.09	-0.6, 0.4	0.72
Re-use	-3×10^{1}	$(-3, 3)\times10^{3}$	0.85	8×10^{2}	$(-3, 5)\times10^{2}$	0.63	-1×10^{-1}	$(-2, 0.3)\times10^{-1}$	0.13	4×10^{4}	$(-3, 9)\times10^{4}$	0.17	2	-2, 7	0.31	1	-2, 4	0.42
Sampling time points	-4×10^{2}	$(-1, 0.1)\times10^{3}$	0.12	-1×10^{2}	$(-7, 4)\times10^{2}$	0.59	-2×10^{-1}	$(-4, 0.5)\times10^{-1}$	0.12	-6×10^{4}	$(-2, 0.5)\times10^{5}$	0.27	-1	-9, 6	0.79	3	-2, 8	0.18
Cell density	3×10^{-3}	$(-0, 5)\times10^{-3}$	0.05	2×10^{-3}	$(-7, 5)\times10^{-3}$	0.13	-1×10^{-7}	$(-1, 1)\times10^{-6}$	0.83				3×10^{-5}	$(-0.9, 7)\times10^{-5}$	0.12	9×10^{-6}	$(-2, 4)\times10^{-5}$	0.49
Nuclei Area	1×10^{1}	$(-2, 5)\times10^{1}$	0.39	-2×10^{1}	$(-5, 2)\times10^{1}$	0.40	1×10^{-2}	$(-0, 3)\times10^{-2}$	0.05	5×10^{3}	$(-0.2, 1)\times10^{4}$	0.12				0.01	-0.3, 0.3	0.93
ZO-1 Area	4	$(-5, 6)\times10^{1}$	0.89	-2×10^{1}	$(-8, 4)\times10^{1}$	0.42	9×10^{-3}	$(-1, 3)\times10^{-2}$	0.41	4×10^{3}	$(-0.7, 1)\times10^{4}$	0.49	0.03	-0.8, 0.9	0.93			
TEER before assay				2×10^{-2}	$(-3, 3)\times10^{-1}$	0.75	-8×10^{-5}	$(-2, 0.2)\times10^{-4}$	0.11	2×10^{1}	$(-4, 8)\times10^{1}$	0.34	2×10^{-3}	$(-2, 6)\times10^{-3}$	0.41	3×10^{-4}	$(-2, 3)\times10^{-3}$	0.77
TEER after assay							4×10^{-5}	$(-2, 2)\times10^{-4}$	0.66	7×10^{1}	$(-0.2, 2)\times10^{2}$	0.14	-3×10^{-3}	$(-1, 0.4)\times10^{-3}$	0.40	-2×10^{-3}	$(-6, 3)\times10^{-3}$	0.42

References

1. Paketuryte, V.; Petrauskas, V.; Zubriene, A.; Abian, O.; Bastos, M.; Chen, W.Y.; Moreno, M.J.; Krainer, G.; Linkuviene, V.; Sedivy, A.; et al. Uncertainty in protein-ligand binding constants: asymmetric confidence intervals versus standard errors. *Eur. Biophys. J. Biophys. Lett.* **2021**, *50*, 661-670, doi:10.1007/s00249-021-01518-4.
2. Delie, F.; Rubas, W. A human colonic cell line sharing similarities with enterocytes as a model to examine oral absorption: Advantages and limitations of the Caco-2 model. *Critical Reviews in Therapeutic Drug Carrier Systems* **1997**, *14*, 221-286.
3. Hidalgo, I.J. Assessing the absorption of new pharmaceuticals. *Current topics in medicinal chemistry* **2001**, *1*, 385-401, doi:10.2174/1568026013395010.
